# Temporal Quantitative Proteomics Reveals Proteomic and Phosphoproteomic Alterations Associated with Adaptive Response to Hypoxia in Melanoma Cells

**DOI:** 10.3390/cancers13092175

**Published:** 2021-04-30

**Authors:** Keshava K. Datta, Parthiban Periasamy, Sonali V. Mohan, Rebekah Ziegman, Harsha Gowda

**Affiliations:** 1 Department of Genetics and Computational Biology, QIMR Berghofer Medical Research Institute, Brisbane, QLD 4006, Australia; Parthiban.Periasamy@qimrberghofer.edu.au (P.P.); Sonali.Mohan@qimrberghofer.edu.au (S.V.M.); Rebekah.Ziegman@qimrberghofer.edu.au (R.Z.); 2 Faculty of Medicine, University of Queensland, Brisbane, QLD 4006, Australia; 3 School of Biomedical Sciences, Queensland University of Technology, Brisbane, QLD 4006, Australia

**Keywords:** mass spectrometry, proteome, phosphoproteome, kinome, proteasome

## Abstract

**Simple Summary:**

Most solid tumours, including melanoma (skin cancer), are riddled with areas lacking adequate oxygen supply due to insufficient vasculature. Cancer cells in these regions are resistant to therapies and contribute to cancer spread and poor treatment response in patients. Understanding the mechanisms by which cancer cells adapt to survive in such a hostile environment will provide novel avenues for treatment. In this study, we investigated mechanisms that melanoma cells use to adapt and survive in an oxygen-poor environment. We used four different melanoma cell lines and studied how protein levels and phosphorylation patterns on thousands of proteins change when the cells are exposed to poor oxygen conditions. This revealed potential mechanisms on which cancer cells are dependent for survival. These survival mechanisms can be potentially targeted to achieve durable response to therapy. We demonstrate this by targeting one such mechanism required for cancer cell survival.

**Abstract:**

Hypoxia is a common feature in various solid tumours, including melanoma. Cancer cells in hypoxic environments are resistant to both chemotherapy and radiation. Hypoxia is also associated with immune suppression. Identification of proteins and pathways that regulate cancer cell survival in hypoxic environments can reveal potential vulnerabilities that can be exploited to improve the efficacy of anticancer therapies. We carried out temporal proteomic and phosphoproteomic profiling in melanoma cell lines to identify hypoxia-induced protein expression and phosphorylation changes. By employing a TMT-based quantitative proteomics strategy, we report the identification and quantitation of >7000 proteins and >10,000 phosphosites in melanoma cell lines grown in hypoxia. Proteomics data show metabolic reprogramming as one of the prominent adaptive responses in hypoxia. We identify several novel hypoxia-mediated phosphorylation changes that have not been reported before. They reveal kinase signalling pathways that are potentially involved in modulating cellular response to hypoxia. In addition to known protein expression changes, we identify several novel proteomic alterations associated with adaptive response to hypoxia. We show that cancer cells require the ubiquitin–proteasome system to survive in both normoxia and hypoxia. Inhibition of proteasome activity affects cell survival and may provide a novel therapeutic avenue to target cancer cells in hypoxia. Our study can serve as a valuable resource to pursue novel candidates to target hypoxia in cancers and improve the efficacy of anticancer therapies.

## 1. Introduction

Hypoxia is a common feature in various solid tumours, including melanoma [1,2]. According to some estimates, 20–30% of cancer cells in a tumour are in a hypoxic environment [3]. Melanoma cells in hypoxic environments are resistant to therapy (chemotherapy and radiation) and are known to display an aggressive phenotype [4]. There is evidence to suggest that the hypoxic environment induces an epithelial to mesenchymal transition (EMT) phenotype and supports growth and maintenance of cancer stem cells. Recent findings also indicate that hypoxic regions in tumours create an immunosuppressive environment promoting immune evasion and may adversely affect the efficacy of immune therapies [5]. Adaptive response to hypoxia is mediated through hypoxia inducible factors (HIFs), a family of transcription factors that regulate the expression of hypoxia-responsive genes. HIF protein stability is negatively regulated by prolyl hydroxylase domain-containing protein EGLN1, which hydroxylates HIF1α and mediates VHL-dependent ubiquitination and subsequent degradation [6,7,8]. Elevated expression of HIFs is associated with increased mortality in various cancers [9,10,11,12]. Since the identification of HIFs as key regulators of hypoxia response, several inhibitors of the HIF pathway have been developed [13]. Examples include actiflavine, a small-molecule drug that binds to HIF1α and HIF1β and inhibits their dimerization and subsequent transcriptional activity, known to show growth arrest in prostate and colorectal cancer models [14,15]. In triple-negative breast cancers, co-administration of HIF inhibitors has been shown to overcome HIF-mediated therapy resistance and immune evasion capability of cancer cells [16,17]. HIF inhibition is also known to block metastatic niche formation in orthotopic models of breast cancer [18]. HIF inhibitors have been shown to target cancer stem cells in leukemia and glioblastoma [19,20]. Despite this experimental evidence and preclinical data, no HIF inhibitors have been approved for treatment due to safety or limited therapeutic efficacy, although >25 inhibitors of HIFs have been tested in clinical trials [13,21].

The significance of targeting hypoxia in cancer therapy is well understood. However, most efforts to target hypoxia are directed at HIFs. As these efforts have not been successful so far, it is important to identify novel candidates to target hypoxia in cancer therapy. Recent studies have revealed various HIF-independent mechanisms associated with oxygen sensing and regulating cell survival in hypoxia [22]. An unbiased investigation to map global proteomic and phosphoproteomic alterations that regulate adaptive response to hypoxia may help identify proteins and pathways that regulate adaptive response in cancer cells. This, in turn, can reveal novel dependencies that can be therapeutically targeted. Targeting hypoxia along with standard of care treatment has the potential to improve therapeutic efficacy and achieve durable response. 

Hypoxia is known to accelerate malignant transformation and cancer progression in melanoma [23,24,25,26]. It is known to promote metastasis and an aggressive phenotype. It is also known to promote vemurafenib resistance in melanoma [27,28]. In this study, we subjected four different melanoma cell lines with mutant BRAF (B-Raf proto-oncogene, serine/threonine kinase) to hypoxia for different durations of time. We carried out temporal proteomics and phosphoproteomics to identify proteomic changes that mediate adaptive response to hypoxia. In addition to known proteins and pathways, we report several novel observations, which can serve as novel avenues for therapeutic targeting of hypoxia in cancers. 

## 2. Results

### 2.1. Temporal Analysis Reveals Altered Protein Expression Pattern in Melanoma Cells Exposed to Hypoxia

**Figure 1:**
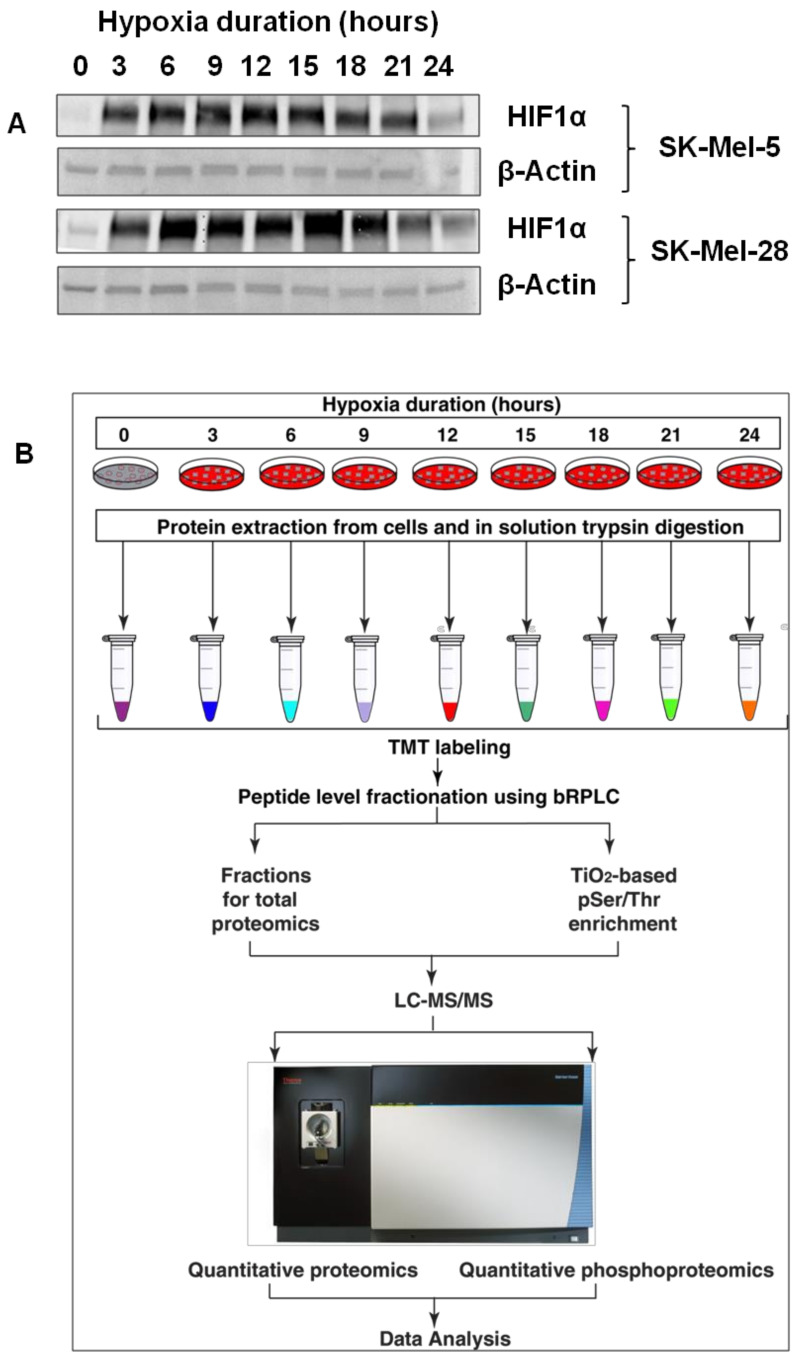
(**A**) Western blotting of HIF1α after exposing cells to different durations of hypoxia. (**B**) Workflow followed for carrying out temporal quantitative proteomic and phosphoproteomic analyses.

We evaluated the expression pattern of hypoxia inducible factor 1 subunit alpha (HIF1A) in four different melanoma cell lines. Melanoma cell lines SK-Mel-5, SK-Mel-28, A2058 and HT-144 were grown under normoxic conditions and transferred to a hypoxia incubator maintained at 1% oxygen for 3, 6, 9, 12, 15, 18, 21 and 24 h, respectively. We carried out Western blotting for HIF1A expression across all time points. HIF1A band was detectable within three hours of transferring cells to hypoxia (Figure 1A, Figure S1). HIF1A levels peaked between 12 and 18 h after exposing cells to hypoxia and declined by 24 h. This is in agreement with most prior observations [29]. In order to characterize temporal changes in the global protein expression pattern, we carried out quantitative proteomics comparing the aforementioned time points. We employed a tandem mass tags (TMT)-based approach to multiplex protein samples from all time points and carried out quantitative proteomic analysis (Figure 1B).

**Figure 2:**
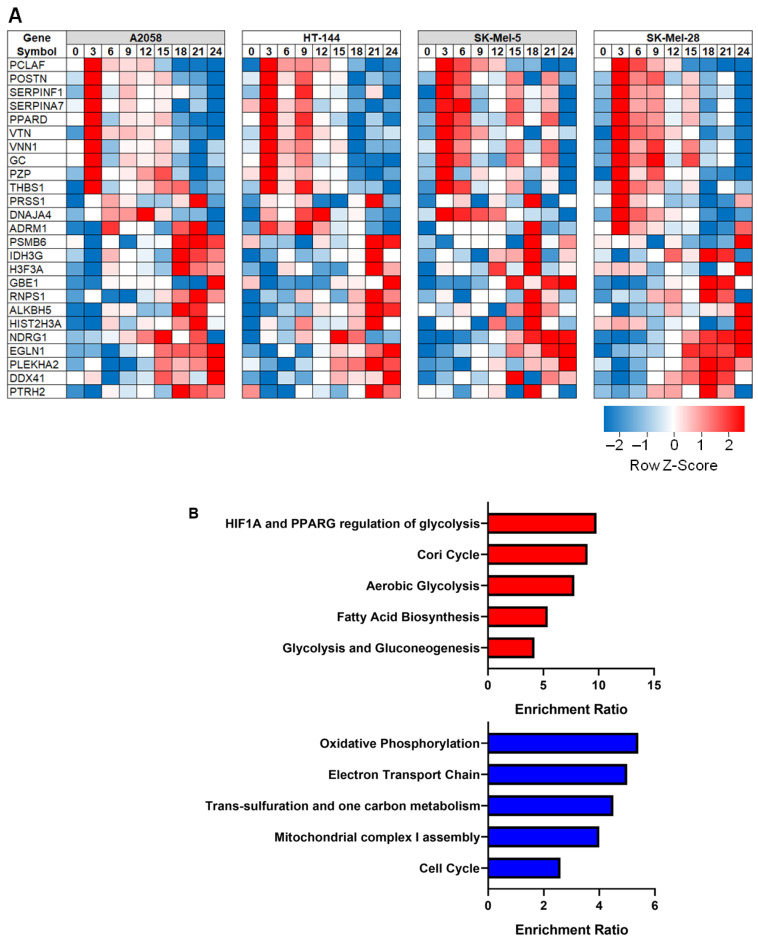
(**A**) Heatmap of proteins that showed hypoxia-responsive expression patterns in all four melanoma cell lines. (**B**) Gene Ontology analysis of dysregulated proteins in hypoxia. Red bars represent overexpressed proteins, and blue bars represent downregulated proteins.

We identified and quantified 7117 proteins from the four cell lines used in this study. Proteins identified in each cell line and associated quantitation data are provided in Table S1. As expected, hypoxia response was heterogeneous across the four cell lines. However, there were also conserved response patterns that were consistent across different cell lines. In order to define conserved hypoxia response patterns across different cell lines, proteins that were differentially regulated in at least two cell lines were considered hypoxia-responsive proteins. These differentially expressed proteins are tabulated in Table S2. Proteomics data showed two distinct responses when cancer cells mounted an adaptive response to hypoxia. Some proteins showed overexpression within 3–6 h after cells were transferred to hypoxia, while several others showed overexpression at later time points. We generated a heatmap for those proteins that displayed differential expression in all four cell lines (Figure 2A).

We carried out Gene Ontology enrichment analysis of differentially regulated proteins (Figure 2B) and found several metabolic pathways to be differentially regulated in hypoxia. While proteins that play a role in glycolysis, Cori cycle, gluconeogenesis and fatty acid biosynthesis were found to be overexpressed in hypoxia, proteins that are part of electron transport chain and oxidative phosphorylation were found to be downregulated in hypoxia. We explore this phenomenon, termed metabolic reprogramming, in greater detail in the next section. 

### 2.2. Metabolic Reprogramming Is a Hallmark of Adaptive Response to Hypoxia

One of the hallmarks of hypoxia response is metabolic reprogramming [30]. Under normoxic conditions, glucose is converted to pyruvate through the glycolytic pathway. Pyruvate then enters the citric acid cycle in mitochondria, generating the reducing equivalents NADH and FADH_2_. These reducing equivalents in turn use the oxidative phosphorylation pathway (OXPHOS) to generate ATP. Aerobic respiration of glucose catabolism is a highly efficient and regulated process generating >30 molecules of ATP for every molecule of glucose. However, during hypoxia, due to lack of oxygen required for OXPHOS, cells switch to anaerobic respiration where the sole source of ATP is the glycolytic pathway. As glycolysis yields only two molecules of ATP for every molecule of glucose catabolized, anaerobic respiration is an inefficient process. As a result, cells experiencing hypoxia need large quantities of glucose to survive.

**Figure 3:**
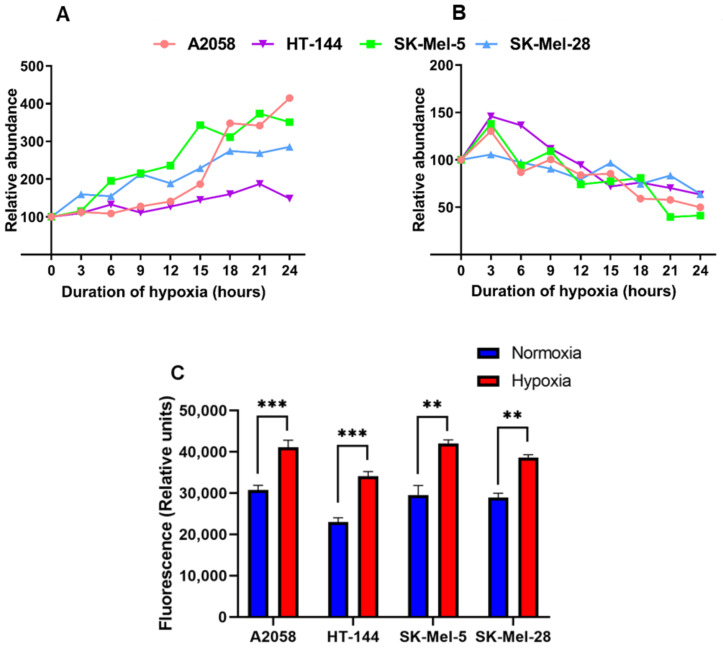
Metabolic reprogramming in hypoxia. (**A**) Expression of SLC2A1 (GLUT1) in hypoxia. (**B**) Expression of ATP5ME in hypoxia. (**C**) Quantification of L-lactate in normoxia and hypoxia. Bars represent mean with SEM (*n* = 3). (** *p* ≤ 0.01; *** *p* ≤ 0.001).

Proteomics data showed differential regulation of several proteins involved in the catabolism of glucose. Glucose is imported into cells by glucose transporters (GLUTs). We observed overexpression of SLC2A1 (GLUT1) with increasing duration of hypoxia (Figure 3A). This helps in the import of more glucose, which is necessary to cope with the energy deficit that is a result of anaerobic breakdown of glucose. Pyruvate, the end product of glycolysis, is converted to lactate in the absence of oxygen. We observed overexpression of lactate dehydrogenase (LDHA), the enzyme that is responsible for this conversion. Metabolic switch to glycolysis was accompanied by downregulation of several components of the OXPHOS complex, including ATP5ME (Figure 3B), NDUFA6, NDUFB5, NDUFB6 and NDUFS8. This was further validated by quantifying L-lactate in normoxia and hypoxia. As expected, L-lactate was significantly higher in hypoxia compared to normoxia (Figure 3C). These data suggest that melanoma cell lines underwent metabolic reprogramming in hypoxia and switched from an OXPHOS phenotype to a glycolytic phenotype. These observations also demonstrate the validity of the hypoxia model used in the current study.

### 2.3. Hypoxia-Induced Phosphoproteomic Alterations Reveal Potential Kinase Signalling Pathways Involved in Adaptive Response to Hypoxia

Post-translational modifications (PTMs) are covalent, reversible, enzymatic modifications of proteins. Protein phosphorylation is one of the most abundant post-translational modifications in cells, which is mediated by more than 500 kinases encoded by the human genome. Reversible protein phosphorylation and dephosphorylation are mediated by protein kinases and protein phosphatases, respectively. Phosphorylation acts as a molecular switch and regulates various cellular processes including proliferation, metabolism, apoptosis, cell cycle, and subcellular protein trafficking and localization [31]. Previous studies have demonstrated the involvement of kinase signalling pathways in regulating hypoxia response. For example, the PI3K signalling pathway is known to regulate HIF expression and is a potential target for cancer therapy [32,33]. In addition, ERBB2 signalling has been shown to increase the rate of HIF1α synthesis [34]. A recent study carried out mitochondrial phosphoproteomics and revealed an AKT–PDK1 signalling axis that mediates metabolic reprogramming in hypoxia [35]. To our knowledge, a temporal phosphoproteomic profiling study to delineate hypoxia-induced phosphorylation changes in cells has not been reported before. We employed a global phosphoproteomic profiling approach to delineate phosphorylation networks that are modulated by hypoxia.

We identified and quantified 13,269 phosphosites corresponding to 2796 proteins. A phosphoRS score of 75% was used as a threshold to identify phosphosites that were mapped reliably with high confidence [36]. Using this stringent threshold, we identified 9456 phosphosites. To our knowledge, this is the most comprehensive phosphoproteomic study in the context of hypoxia in melanoma. Phosphopeptides identified in each cell line and associated quantitation data are provided in Table S3. 

**Figure 4:**
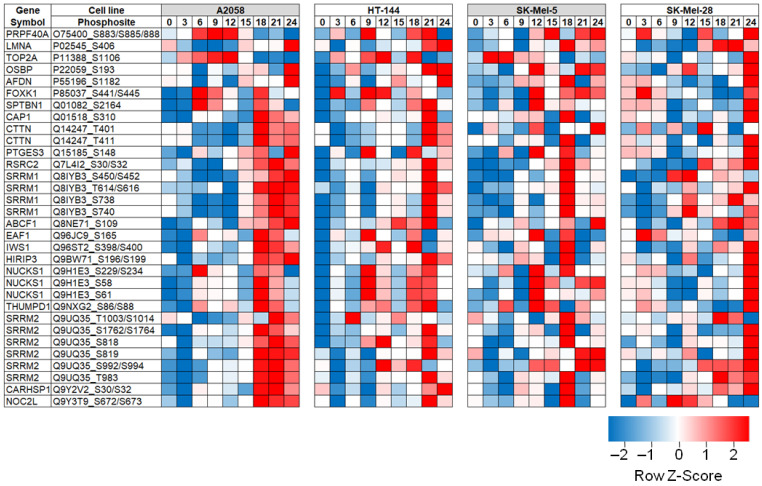
Heatmap of phosphosites that showed hypoxia-responsive phosphorylation patterns in all four melanoma cell lines.

As expected, phosphoproteomic alterations were heterogenous between the four cell lines that were studied. In order to determine conserved hypoxia response across different cells, we considered phosphosites that were differentially phosphorylated in two or more cell lines to be hypoxia-responsive phosphosites. These differentially phosphorylated sites are tabulated in Table S2. Further, there were several site-specific alterations that were highly conserved and were observed in all four cell lines (Figure 4).

In order to determine kinase-driven signalling pathways that potentially modulate phosphoproteomic response, we carried out upstream kinase analysis for the 47 phosphosites that were commonly hyperphosphorylated across all four cell lines using KiNEXUS (http://www.phosphonet.ca/). Mitogen-activated protein kinases (ERK1 and ERK2) and casein kinase were predicted to be upstream kinases for most protein substrates. Interestingly, we identified differentially phosphorylated sites on both ERK1 and ERK2. We found decreased phosphorylation of ERK1 at T207, which is an inhibitory site [37], indicating activation of ERK1. ERK1 activation was corroborated by hyperphosphorylation of several predicted substrates, including serine and arginine repetitive matrix 2 (SRRM2) at T983, S994 and S1014; cortactin (CTTN) at T411; and spectrin beta chain, brain 1 (SPTBN1) at S2164.

ERK2 was hyperphosphorylated in hypoxia at T190, a known activation site [38]. In hypoxia, we found hyperphosphorylation of serine and arginine repetitive matrix 1 (SRRM1) at S452 and S616; and forkhead box K1 (FOXK1) at S445. These predicted ERK1/2 phosphorylation sites provide potential mechanistic insights into ERK1/2-mediated signalling response in hypoxia.

We found decreased phosphorylation of casein kinase 1 epsilon (CSNK1E) at S408, an inhibitory site [39], indicating activation. We identified hyperphosphorylation of DNA topoisomerase 2-alpha (TOP2A) at S1106 in hypoxia, a known substrate of CSNK1E [40].

Overall, this dataset provides a number of kinases and potential substrates that are modulated in response to hypoxia. These kinase pathways can be potentially targeted to inhibit adaptive response of cancer cells to hypoxia.

### 2.4. The Ubiquitin–Proteasome System Is Elevated When Cells Mount an Adaptive Response to Hypoxia

**Figure 5:**
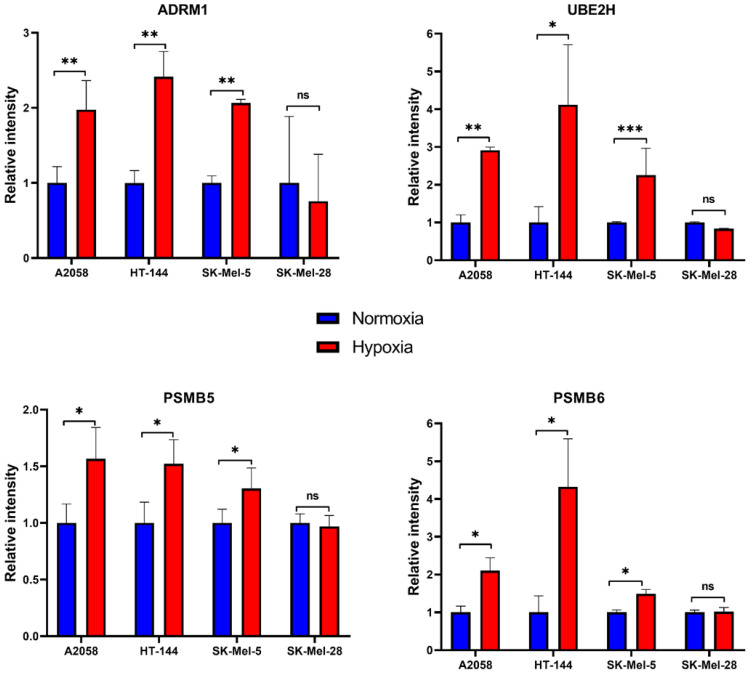
Targeted proteomics to validate the overexpression of a subset of proteins involved in the ubiquitin–proteasome degradation pathway. Cells were exposed to normoxia (21% oxygen) or hypoxia (1% oxygen) for 24 h, harvested and prepared for proteomic analysis (*n* = 3). Bars represent mean with SEM (*n* = 3). (ns = not significant (*p* > 0.05); * *p* ≤ 0.05; ** *p* ≤ 0.01; *** *p* ≤ 0.001).

A recent study by Jain et al. [41] reported genome-wide CRISPR growth screens to identify genes that affect fitness in normoxic and hypoxic conditions. We compared the list of proteins from our study that showed hypoxia-induced expression patterns with the list of genes reported to affect fitness in hypoxic conditions, by genetic screen. There were 180 genes common to the two lists (Table S4). As the cell lines used in these two studies are from different cancers, common genes represent potentially conserved mechanisms that are employed by cells to mount an adaptive response to hypoxia. Among common members, we observed several proteins that belonged to the proteasome degradation pathway. Overexpression of components of the proteasome degradation pathway included adhesion regulating molecule 1 (ADRM1), which is a proteasomal ubiquitin receptor, and catalytic subunits of proteasome, proteasome subunit beta type-5 (PSMB5) and proteasome subunit beta type-6 (PSMB6). We also observed overexpression of derlin 1 (DERL1) in hypoxia. Derlin family proteins play an important role in endoplasmic reticulum (ER)-associated stress response by translocating proteins from the ER to the cytosol for ubiquitin-mediated proteasome degradation under ER stress conditions [42]. Previous reports have shown that DERL1 is overexpressed in breast cancers and protects cancer cells from ER stress-induced apoptosis [43]. Overexpression of DERL1 is associated with poor survival in head and neck squamous cell carcinoma [44]. Interestingly, proteins belonging to the ubiquitin pathway, which is upstream of proteasome degradation, were also overexpressed in hypoxia. This included ubiquitin-conjugating enzymes such as ubiquitin-conjugating enzyme E2 L3 (UBE2L3), ubiquitin-conjugating enzyme E2 H (UBE2H) and ariadne RBR E3 ubiquitin protein ligase (ARIH2). We also observed overexpression of ring finger protein 14 (RNF14), which is suspected to act as an ubiquitin ligase in the ubiquitination of nuclear proteins [45]. We employed targeted proteomics to validate overexpression of a subset of proteins of the ubiquitin–proteasome pathway (Figure 5).

### 2.5. Proteasome Activity Is Essential for Cancer Cells to Survive in Hypoxia

**Figure 6:**
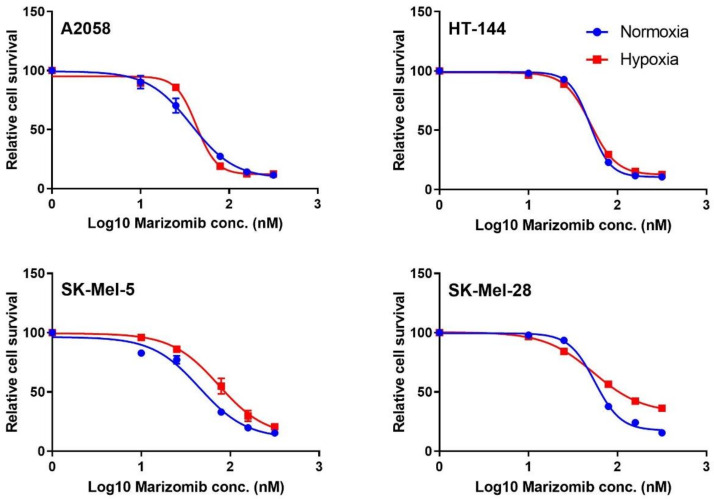
Proteasome inhibition affects cancer cell survival in hypoxia and normoxia. Cell viability of melanoma cell lines in response to different concentrations of marizomib is shown. Data points represent mean with SD (*n* = 3).

Based on data from a recently published genetic screen to identify genes that affect fitness in hypoxia and our own data that identified proteins showing hypoxia-induced expression patterns, we reasoned that cancer cells depend on the proteasome degradation pathway for their survival in hypoxia. In order to determine if cancer cells show dependency on proteasome activity, we treated all four melanoma cell lines with marizomib, a potent inhibitor of 20S proteasome activity, under normoxic and hypoxic conditions. All four cell lines were sensitive to proteasome inhibition (Figure 6). In order to evaluate if proteasome dependency is melanoma-specific, we treated lung and breast cancer cell lines in normoxia and hypoxia with marizomib (Figure S2). Proteasome activity was essential for survival across all cancer cell lines in both hypoxia and normoxia.

**Figure 7:**
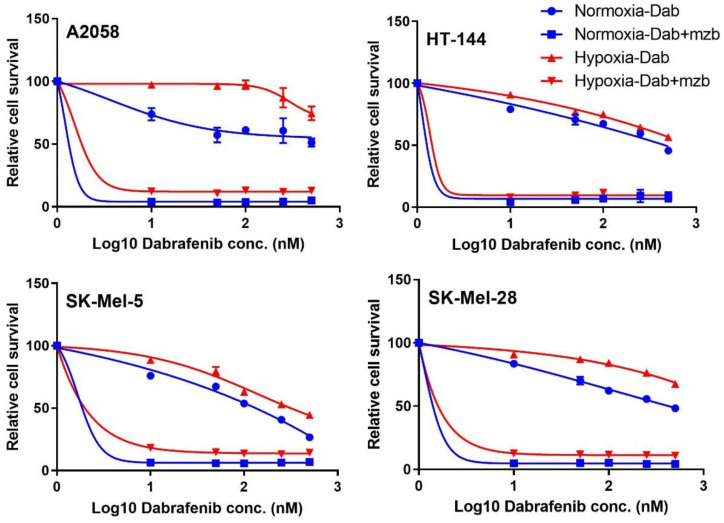
Combined treatment with dabrafenib (dab) and marizomib (mzb) affects survival of melanoma cells in hypoxia and normoxia. Cell viability of the four cell lines A2058, HT-144, SK-Mel-5 and SK-Mel-28 in response to dabrafenib treatment and combination of dabrafenib and marizomib is shown. Dabrafenib treatment was done at the indicated doses. For combination treatment, 100 nM marizomib was used along with different concentrations of dabrafenib. Data points represent mean with SD (*n* = 3).

Hypoxia is associated with drug resistance. All four melanoma cell lines used in the study harbour activating BRAF mutations. We treated melanoma cell lines with increasing doses of dabrafenib, a mutant BRAF inhibitor used for treating melanoma, as a single agent and in combination with marizomib. The cells showed potent response in combination with marizomib (Figure 7). Based on these observations, proteasome activity appears to be vital for cancer cell adaptation to hypoxia. As this dependency was observed for different cancer types, proteasome inhibition may be effective as a pan-cancer target for hypoxia. 

## 3. Discussion

Molecular oxygen is necessary to sustain various intracellular biochemical reactions and to maintain energy homeostasis. Therefore, a decreased concentration of oxygen (hypoxia) is a major stress factor, and cells have evolved mechanisms to mount an adaptive response to cope with low oxygen stress. Solid tumours often have abnormal vasculature, which leads to severe constraints on oxygen and nutrient diffusion to cancer cells. This creates regions with effective vasculature that are normoxic and regions with poor vasculature that experience mild to severe hypoxia, or necrotic regions with dead cells due to lack of nutrients and oxygen. Hypoxia is known to induce EMT, support growth and maintenance of cancer stem cells and promote metastasis. Hypoxia is associated with drug resistance and is known to create an immunosuppressive environment, which affects the efficacy of immune therapies. Therefore, tumour hypoxia is often associated with relapse. These observations provide a strong rationale for targeting cancer cells in hypoxia to improve the efficacy of cancer therapies. 

The most widely studied mechanism of hypoxic response is that of the HIFs (hypoxia inducible factors). Although these transcription factors are constitutively expressed, they are stabilized only under hypoxic conditions and control the expression of several hypoxia-responsive genes. HIFs are regulated by hydroxylation of proline residues by prolyl hydroxylases. EGLN1 is a member of the prolyl hydroxylase family, which consists of three members. Localized in the cytoplasm, EGLN1 hydroxylates proline residues on target proteins, specifically HIF1α, and mediates VHL-dependent ubiquitination and subsequent degradation [46]. EGLN1 is known to be overexpressed in a number of cancers [47]. It is a known oxygen sensor and was overexpressed in hypoxia in all the four cell lines that we studied. 

Since the discovery of HIFs as key mediators of response to hypoxia, most studies have focused on the role played by HIFs and their targets. However, several studies have now shown HIF-independent mechanisms involved in mounting an adaptive response to hypoxia [48]. Over the years, several small-molecule inhibitors have been developed to target hypoxia in cancers. Despite favourable response in preclinical studies, there is no effective inhibitor approved for targeting hypoxia in cancers. Most pan-HIF inhibitors that are promising in preclinical studies have dose-limiting side effects that prevent their use for therapy. Better understanding of proteins and pathways that modulate cellular response to hypoxia is necessary to identify novel therapeutic targets to achieve durable response to therapies and mitigate hypoxia-mediated drug resistance. 

There are a few studies that have carried out proteomic analysis of cancer cell lines in hypoxia. Ross et al. utilized PC-3, a hormone-insensitive prostate cancer cell line, and employed data-independent acquisition-based proteome analysis in hypoxia [49]. Song et al. utilized two isogenic osteosarcoma cell lines with different metastatic propensities and carried out label-free shotgun proteomic analysis to delineate the hypoxia-induced proteome [50]. In the context of melanoma, Walbrecq et al. carried out miRNome and proteome profiling of four melanoma cell lines in hypoxia [51]. Hypoxia-mediated proteomic changes have also been investigated in breast cancer [52], cervical cancer [53] and glioblastoma [54]. A comparative analysis of our study with these studies shows that a metabolic switch driven by the overexpression of components of the glycolytic pathway and downregulation of proteins belonging to the oxidative phosphorylation and tricarboxylic acid cycle is a conserved response to hypoxia across different systems. In addition, the protein hydroxylation pathway is a core response to hypoxia, mostly driven by proteins such as EGLN1, P4HA1 and P4HA2. Interestingly, the overexpression of the proteasome-mediated protein degradation pathway that we observed has also been reported in glioblastoma. These commonalities may demonstrate tissue-agnostic signatures of tumour hypoxia. 

In this study, we carried out an unbiased investigation of proteins and pathways that are activated in response to hypoxia. By studying four different cancer cell lines, we could identify several conserved responses to hypoxia. We compared the list of overexpressed proteins in response to hypoxia with genes that affect fitness in hypoxia, which were reported by Jain et al. using a genome-wide CRISPR screen. There were 180 genes that were common between the two studies. This orthogonal evidence prompted us to evaluate cancer cell dependency in hypoxia that can be potentially targeted as a therapeutic strategy. Among several processes, ubiquitin-mediated proteasome degradation was found to be one of the essential mechanisms required for adaptive response to hypoxia. The current study also identified several kinases that showed hypoxia-induced expression and/or phosphorylation patterns. Most of these proteins and phosphorylation sites have no prior association with hypoxia or the HIF pathway. This should serve as a valuable resource for the community to develop therapeutic strategies to target cancer cells in hypoxia. 

## 4. Materials and Methods

All chemicals were obtained from Millipore Sigma (Darmstadt, Germany) unless specified.

### 4.1. Cell Culture and Hypoxia Exposure

Melanoma cell lines A2058, HT-144, SK-Mel-5 and SK-Mel-28 were kindly provided by Dr. Glen Boyle (QIMR Berghofer Medical Research Institute, Brisbane, Australia). The cell lines were cultured in RPMI-1640 medium supplemented with 10% fetal bovine serum (FBS). All cell lines were tested for *Mycoplasma* infection and authenticated using short tandem repeat (STR) profiling by scientific services at QIMR Berghofer Medical Research Institute.

Cells were grown at 37 °C in a humidified 5% CO_2_ incubator. For hypoxia exposure, cells were grown to ~75% confluency and shifted to an incubator that maintained 1% oxygen concentration and harvested after different durations—3, 6, 9, 12, 15, 18, 21 and 24 h. Cells were given a quick rinse with ice-cold phosphate buffered saline (PBS), and lysis buffer was added (2% SDS in 100 mM triethyl ammonium bicarbonate buffer with protease and phosphatase inhibitors). The resulting cell lysate was collected in microcentrifuge tubes, sonicated, and clarified by centrifugation. Protein concentration was estimated using the bicinchoninic acid (BCA) assay. 

### 4.2. Western Blotting

HIF1α (Cat. # 36169) and β-actin (Cat. # 4967) antibodies were obtained from Cell Signaling Technology (Danvers, MA, USA). Immunoblotting was carried out as described earlier [55]. The chemiluminescent ECL Prime (GE Life Sciences, Marlborough, MA, USA) was used to detect target proteins. Detailed information about the western blot can be found at Supplementary Materials.

### 4.3. Protein Digestion and TMT Labelling

Equal amounts of protein samples from all conditions were reduced using 5 mM dithiothreitol and alkylated with 20 mM iodoacetamide. Proteins were precipitated using ice-cold acetone, and in-solution trypsin digestion of samples was carried out using a 1:20 enzyme-to-substrate ratio. Trypsin-digested peptides were lyophilized and labelled with tandem mass tags (TMT) as per the manufacturer’s protocol (Thermo Scientific, Waltham, MA, USA). Briefly, the TMT labels were brought to room temperature and dissolved in 41 µL anhydrous acetonitrile. The lyophilized peptides were reconstituted in 100 mM TEAB buffer, mixed with the TMT reagent and incubated for one hour at room temperature. The reaction was quenched by adding 8 µL of 5% hydroxyl amine and incubating for fifteen minutes at room temperature. TMT-labelled peptides were pooled and subjected to basic pH reverse phase liquid chromatography (bRPLC)-based fractionation [56]. After concatenation, a total of 12 fractions were obtained. Each fraction was split into 10% (for total proteome analysis) and 90% (for phosphoproteomic analysis) and lyophilized. 

### 4.4. Phosphopeptide Enrichment

Phosphopeptide enrichment was carried out as described before [57] with slight modifications. Peptides were reconstituted in 300 µL of 100 mM TEAB, mixed with 400 µL isopropanol and incubated on a thermoshaker (1400 RPM, 3 min, RT). Then, 100 µL of enrichment buffer (48% TFA, 8 mM KH_2_PO_4_) was added, followed by incubation on a thermoshaker (1400 RPM, 3 min, RT). The tubes were centrifuged (21,000× *g*, 5 min, RT) to ensure no pellet was formed. TiO_2_ (GL Sciences Inc., Tokyo, Japan) was resuspended in loading buffer (6% TFA, 80% ACN) and added to the tubes at a ratio of 1:12 (peptide:TiO_2_ *wt*/*wt*). The tubes were incubated on a thermoshaker (1400 RPM, 20 min, 40 °C). At the end of incubation, the tubes were centrifuged (2000× *g*, 2 min, RT) and the supernatant was discarded. Then, 500 µL of wash buffer (5% TFA, 60% isopropanol) was added to each tube, followed by incubation on a thermoshaker (1400 RPM, 3 min, RT). The tubes were centrifuged (2000× *g*, 2 min, RT), and the supernatant was discarded. The wash step was repeated thrice. Phosphopeptides were eluted with 100 µL of elution buffer (48:32:20 = H_2_O:ACN:NH_4_OH). The elution was facilitated by incubating on a thermoshaker (1400 RPM, 10 min, RT). The tubes were centrifuged (21,000× *g*, 5 min, RT), and the eluate was collected into fresh tubes. The eluate was dried in a speedvac.

### 4.5. Liquid Chromatography–Tandem Mass Spectrometry Analysis

The phosphopeptide-enriched samples as well as the total proteome samples were analysed on an Orbitrap Fusion Tribrid mass spectrometer (Thermo Electron, Bremen, Germany) coupled to a nanoAcquity UHPLC (Waters, Milford, MA, USA) as described earlier [58]. The LC settings for both total proteome and phosphoproteome were identical: The peptide samples were first loaded onto an Acquity UPLC M-Class V/M symmetry trap column (Waters, Milford, MA, USA) at a flow rate of 5 µL/min, and then resolved on an Acquity UPLC M-Class Peptide BEH C18 nanoAcquity column (Waters, Milford, MA, USA) over a runtime of 120 min at a flow rate of 300 nl/min. 

The MS settings for total proteome were: MS1 Resolution—60,000; Mass Range—350–1400 *m*/*z*; AGC Target—1 × e^6^, Maximum injection time 50 ms. Include charge state—2–7; Dynamic exclusion—40 s. MS2: Isolation mode—Quadrupole; Isolation window—0.4; Activation type—CID, Collision energy—35%; AGC target—10,000, Maximum injection time—35 ms. MS3: Isolation window—2; Activation type—HCD, Collision energy—55%; Orbitrap resolution—50,000; AGC target—50,000, Maximum injection time—86 ms.

The MS settings for phosphoproteome were: MS1 Resolution—60,000; Mass Range—350–1800 *m*/*z*; AGC Target—4 × e^5^, Maximum injection time 50 ms. Include charge state—2–6; Dynamic exclusion—90 s. MS2: Isolation mode—Quadrupole; Isolation window—0.7; Activation type—HCD, Collision energy—38%; AGC target—100,000, Maximum injection time—105 ms.

### 4.6. Data Analysis

Raw files obtained from mass spectrometry analysis were searched against the UniProt protein database (Proteome ID—UP000005640; 49,070 sequences; database accessed on 1 July 2018), using Sequest HT through Proteome Discoverer (Version 2.2) (Thermo Scientific, Bremen, Germany). Precursor and fragment mass tolerance were set to 10 ppm and 0.05 Da, respectively. TMT at N-terminus and lysine and carbamidomethylation of cysteine were set as fixed modifications, while oxidation of methionine was set as a dynamic modification. For phosphoproteomic data, phosphorylation at serine, threonine and tyrosine was also set as a dynamic modification. A false discovery rate (FDR) threshold of 1% was used to filter peptide spectrum matches (PSMs). FDR was calculated using a decoy search. For phosphoproteomic data, a phosphoRS score threshold of ≥75% was used for phosphosite localization. Data were normalized based on total peptide amount using the normalization feature available in the “Reporter Ions Quantifier” node of Proteome Discoverer. Proteins that did not have quantification values across all time points (<5% of data) were removed from the dataset. A fold change threshold of 1.5 was used to determine differentially expressed or phosphorylated proteins.

### 4.7. Bioinformatics Analysis

Heatmaps (Figure 2A and Figure 4) of differentially regulated/phosphorylated molecules were generated using Morpheus (https://software.broadinstitute.org/morpheus, accessed on 27 January 2021). Gene Ontology analysis of differentially regulated proteins (Figure 2B) was carried out using WebGestalt—WEB-based GEne SeT AnaLysis Toolkit [59]. Upstream kinase analysis was carried out using PhosphoNet by Kinexus (http://www.phosphonet.ca/, accessed on 20 February 2021).

### 4.8. Lactate Quantification

L-lactate assay kit by Cayman Chemical (Ann Arbor, MI, USA) was employed to quantify lactate, and the experiment was carried out as per the manufacturer’s instructions for extracellular lactate quantification. 

### 4.9. Targeted Proteomics

We carried out targeted proteomic analyses of proteins of the ubiquitin–proteasome pathway. Inclusion lists comprised the peptide *m*/*z* and charge values generated for the proteins of interest using the previously obtained data-dependent analysis. Samples were analysed using the instrument parameters previously described with slight modifications. Briefly, parent peptides were detected in the Orbitrap at a resolution of 60,000, and those that matched the specifications from the inclusion list with a mass tolerance of +/− 10 ppm were selected for MS2. MS2 was performed using HCD with stepped collision energies of 33%, 38% and 43% and an Orbitrap resolution of 60,000. Maximum injection time was 118 ms, while the AGC target was 50,000 and the isolation window was 1.6. Raw data were analysed on Proteome Discoverer 2.2 using default parameters.

### 4.10. MTS Assay

Marizomib (Salinosporamide A) was procured from Cayman Chemical (Ann Arbor, MI, USA) (Cat. # 10007311). Dabrafenib was procured from Selleckchem (Houston, TX, USA) (Cat. # S2807). MTS reagent was procured from Promega (Madison, WI, USA).

Equal numbers of cells were seeded and allowed to adhere overnight. Different concentrations of either marizomib or dabrafenib were added to each well, and the cells were incubated in hypoxia for 72 h. MTS reagent (3-(4,5-dimethylthiazol-2-yl)-5-(3-carboxymethoxyphenyl)-2-(4-sulfophenyl)-2H-tetrazolium) was added, followed by incubation for four hours. Absorbance was recorded at 490 nm. Survival plots were generated using GraphPad Prism version 8.

### 4.11. Statistical Analyses

GraphPad Prism version 8 was used for statistical analyses (GraphPad Software, La Jolla, CA, USA). For both lactate quantification and targeted proteomics, statistical significance was determined using student’s *t*-tests.

### 4.12. Data Availability

Mass spectrometry data generated in this study have been deposited to the ProteomeXchange Consortium through the PRIDE partner repository with dataset identifier PXD019832.

## 5. Conclusions

Hypoxia is known to affect the efficacy of anticancer therapy. Although targeting hypoxia is considered a useful strategy, there are no reliable targets that have been approved for therapeutic targeting of cancer cells in hypoxia. Here, we employed a global proteomic and phosphoproteomic strategy for unbiased characterization of proteins and pathways that are modulated when melanoma cells mount an adaptive response in hypoxic conditions. Our study revealed several proteins whose expression and phosphorylation were affected by hypoxia. We observed overexpression of several proteins associated with the ubiquitin–proteasome system. We reasoned that the proteasome degradation pathway is essential for mounting an adaptive response to hypoxia. Proteasome inhibition affected cell survival in hypoxia. This was not restricted to melanoma as we observed the same effect in breast and lung cancer cells, indicating that it is an essential component. This study provides mechanistic insights into proteins and pathways that play a crucial role in the adaptive response to hypoxia and reveals several novel candidates that could be potentially targeted.

## Data Availability

The data presented in this study are openly available in ProteomeXchange Consortium with dataset identifier PXD019832.

## Supplementary Materials

The following are available online at https://www.mdpi.com/article/10.3390/cancers13092175/s1, Table S1: List of all the proteins identified and quantified in each cell line used in the current study, Table S2: List of proteins and phosphopeptides that showed differential expression/phosphorylation in at least two cell lines, Table S3: List of all the phosphopeptides identified and quantified in each cell line used in the current study, Table S4: List of proteins that showed hypoxia-induced expression and were reported to affect fitness in hypoxia, Figure S1 (Related to Figure 1A): Western blotting of HIF1α after exposing cells to different durations of hypoxia (A2058 and HT-144), Figure S2 (Related to Figure 6): Proteasome inhibition affects breast and lung cancer cell survival in hypoxia and normoxia. Data points represent mean with SD (*n* = 3).

## List of Abbreviations

LCMS—liquid chromatography–mass spectrometry; TMT—tandem mass tags; EMT—epithelial to mesenchymal transition; TEAB—triethyl ammonium bicarbonate.
