# Supplementary material for: Temporal Quantitative Proteomics Reveals Proteomic and Phosphoproteomic Alterations Associated with Adaptive Response to Hypoxia in Melanoma Cells
Source: Cancers (Basel). 2021 Apr 30;13(9):2175. doi: 10.3390/cancers13092175 (PMC8124723; doi:10.3390/cancers13092175)
Supplement: Supplementary file 1 [file cancers-13-02175-s001.zip › cancers-1197037 - supple- proofreading/original image about WB.pdf]

# Western blot: HIF1α. Antibody – CST (#36169)

**SK-Mel-5**

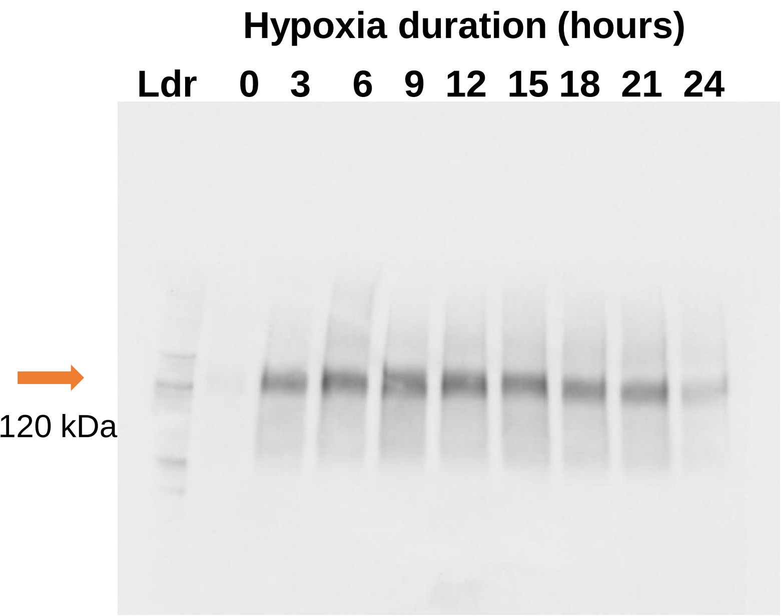


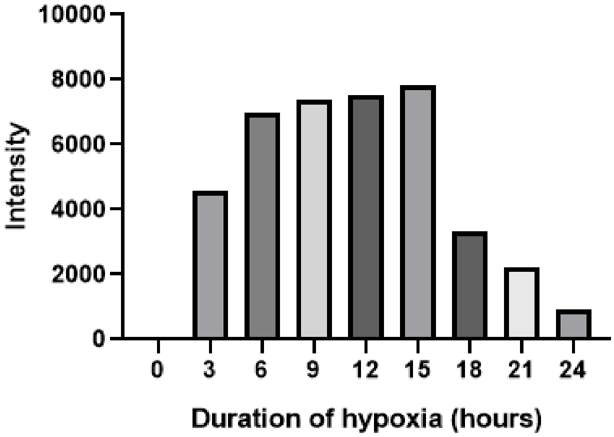


**SK-Mel-28**

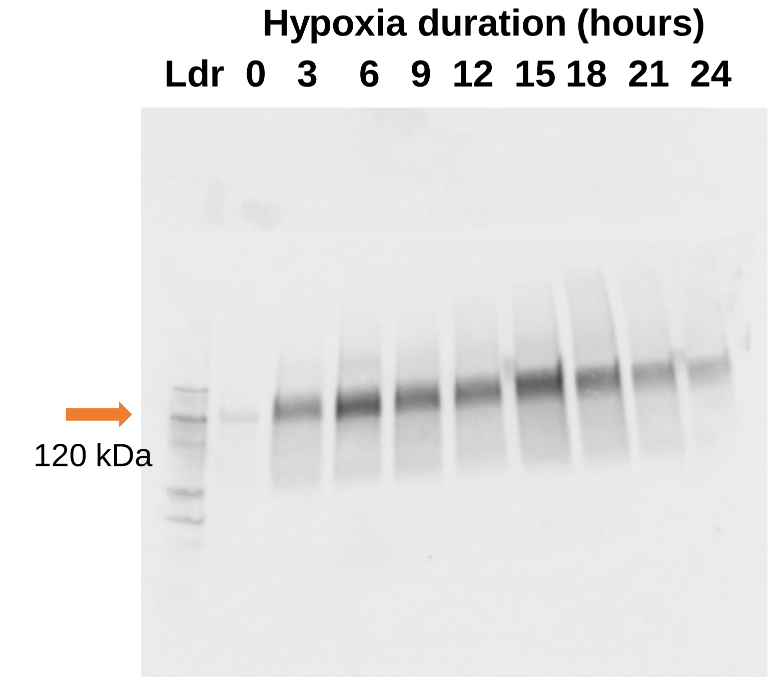


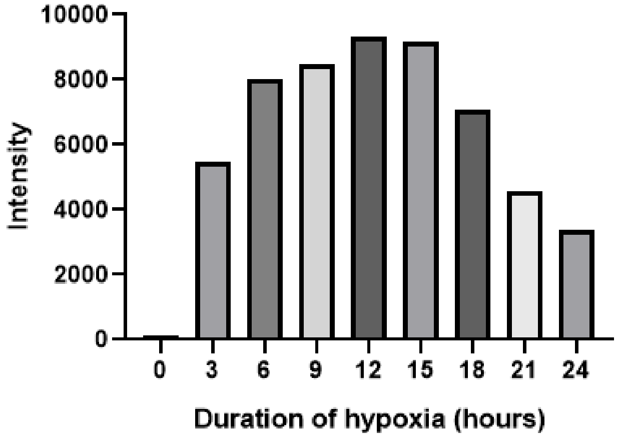


Ldr = Ladder. Biotinylated Protein Ladder Detection Pack (CST #7727)

# Western blot: β-actin. Antibody – CST (#4967)

**SK-Mel-5**

**Hypoxia duration (hours)**

**Ldr 0 3 6 9 12 15 18 21 24**

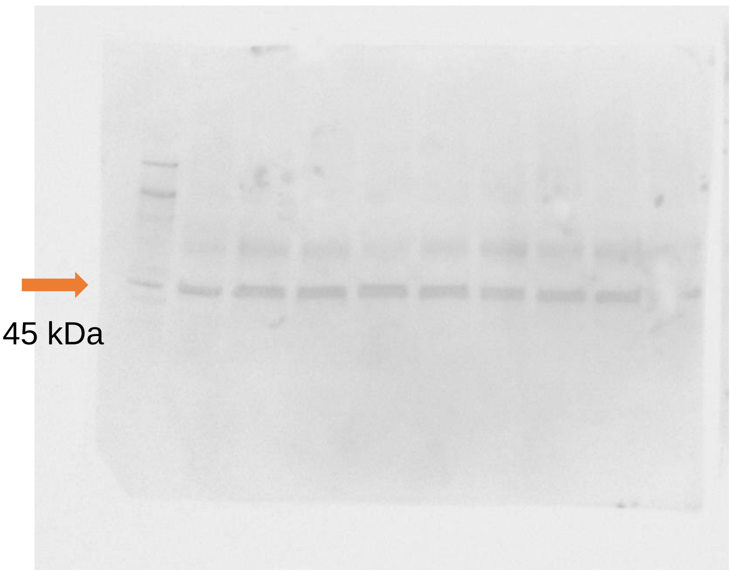


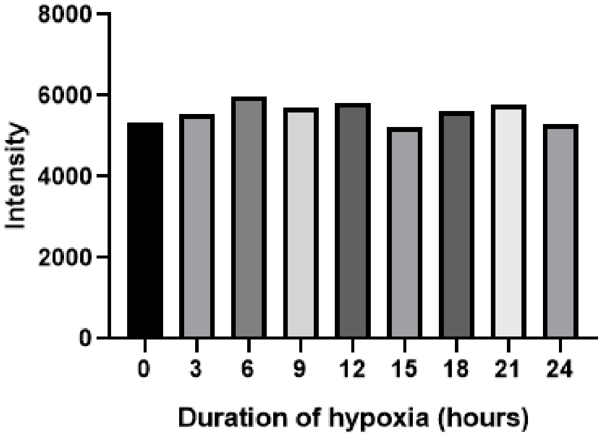


**SK-Mel-28**

**Hypoxia duration (hours)**

**Ldr 0 3 6 9 12 15 18 21 24**

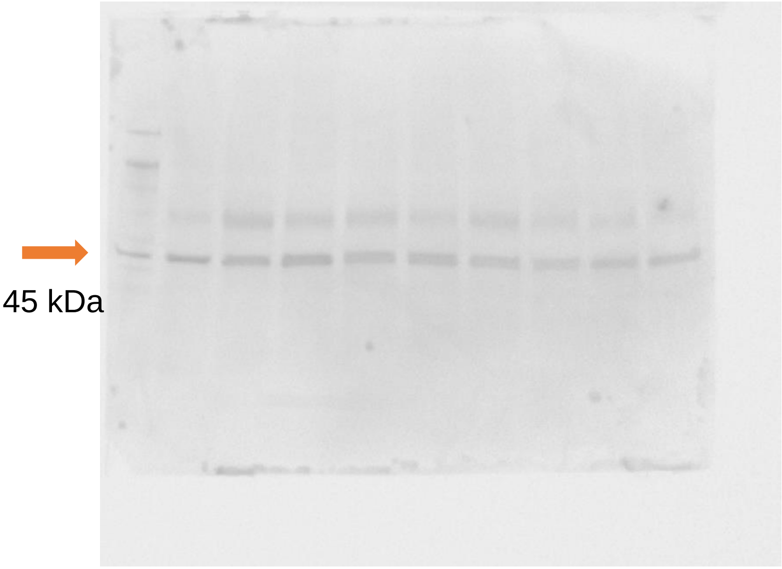


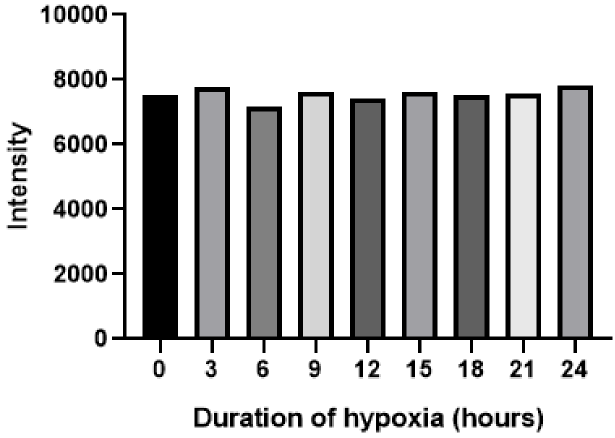


Ldr = Ladder. Biotinylated Protein Ladder Detection Pack (CST #7727)

# Western blot: HIF1α. Antibody – CST (#36169)

**A2058**

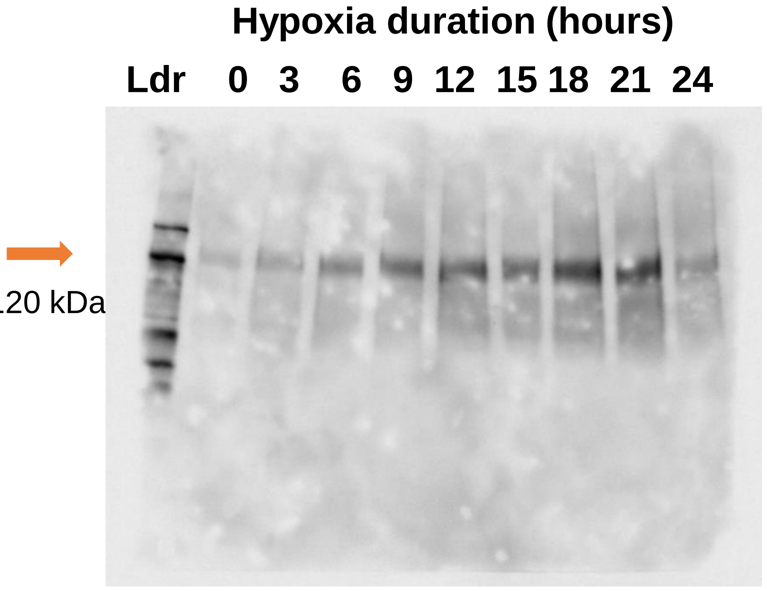


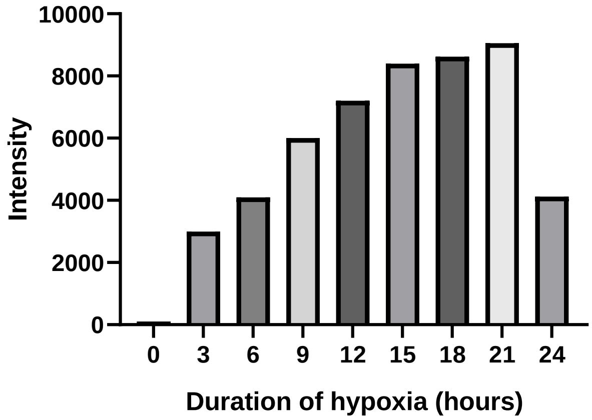


**HT-144**

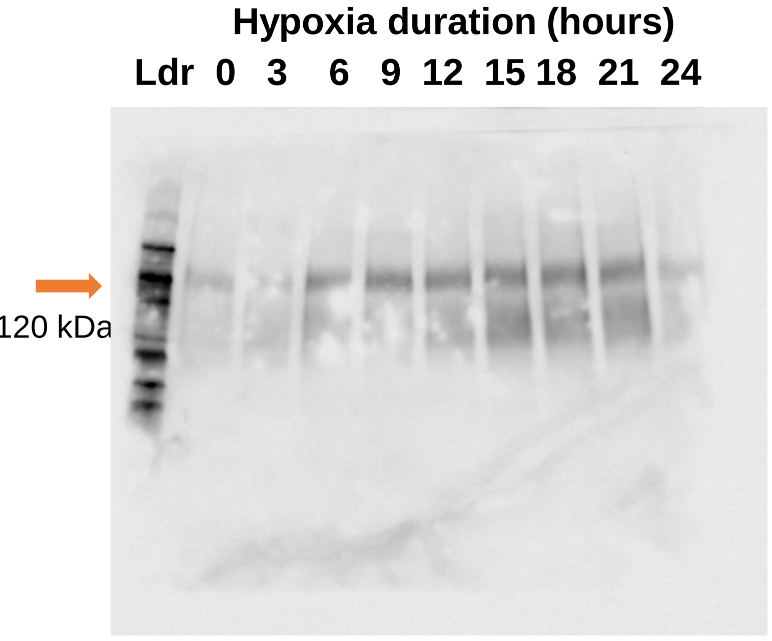


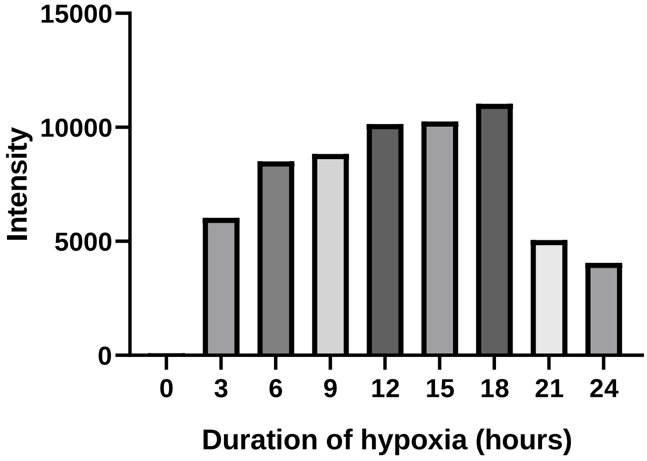


Ldr = Ladder. Biotinylated Protein Ladder Detection Pack (CST #7727)

# Western blot: β-actin. Antibody – CST (#4967)

**A2058**

**Hypoxia duration (hours)**

**Ldr 0 3 6 9 12 15 18 21 24**

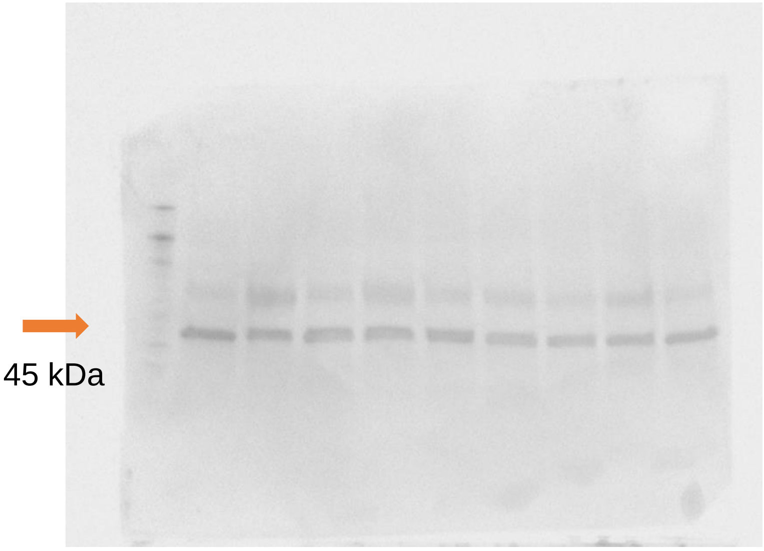


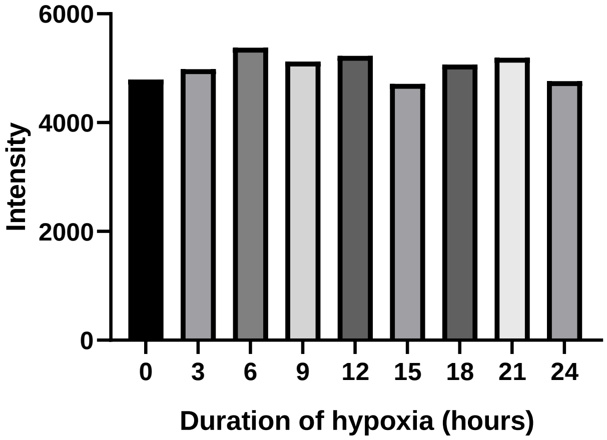


**HT-144**

**Hypoxia duration (hours)**

**Ldr 0 3 6 9 12 15 18 21 24**

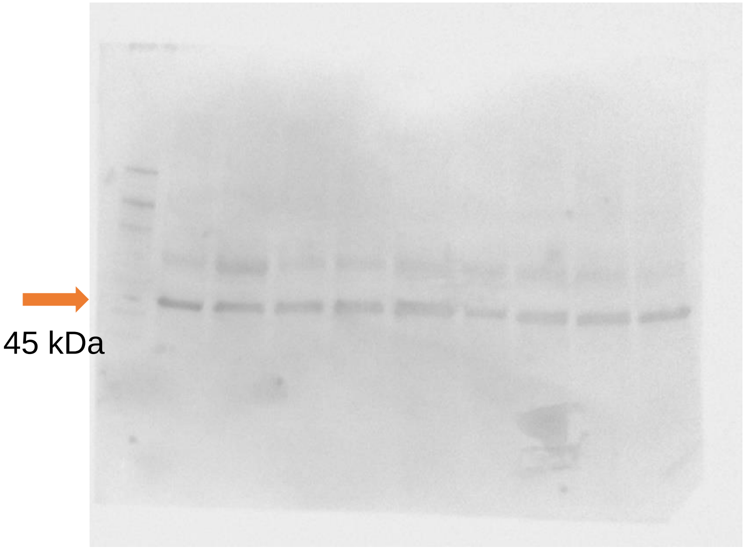


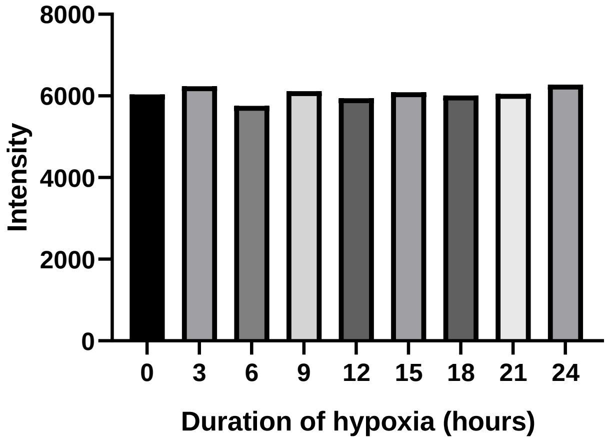


Ldr = Ladder. Biotinylated Protein Ladder Detection Pack (CST #7727)
