# Supplementary material for: Temporal Quantitative Proteomics Reveals Proteomic and Phosphoproteomic Alterations Associated with Adaptive Response to Hypoxia in Melanoma Cells
Source: Cancers (Basel). 2021 Apr 30;13(9):2175. doi: 10.3390/cancers13092175 (PMC8124723; doi:10.3390/cancers13092175)

## Supplementary Figure 1

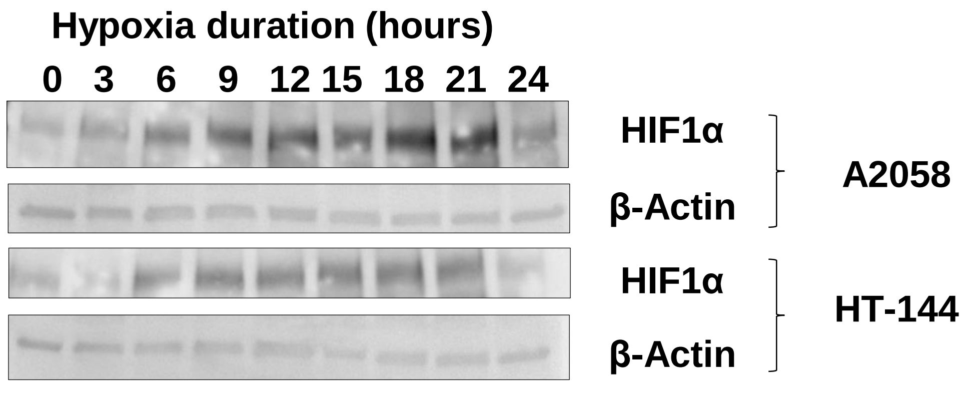

**Supplementary Figure 2**

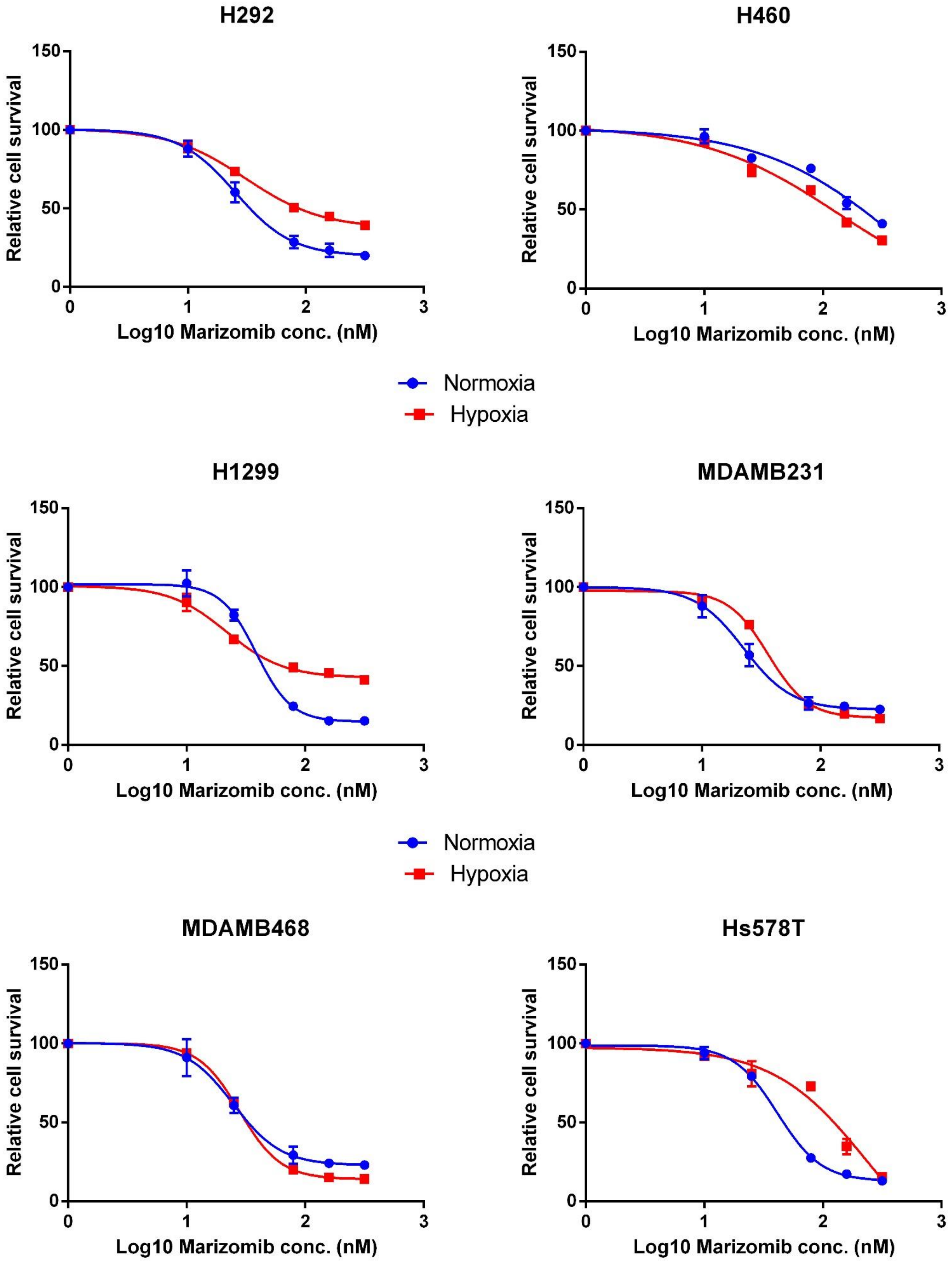

Supplement: Supplementary file 1 [file cancers-13-02175-s001.zip › cancers-1197037 - supple- proofreading/Supplementary_Figures.pdf]
